# Choroid plexus-targeted viral gene therapy for alpha-mannosidosis, a prototypical neurometabolic lysosomal storage disease

**DOI:** 10.1093/hmg/ddae201

**Published:** 2025-01-16

**Authors:** Eun-Young Choi, John H Wolfe, Stephen G Kaler

**Affiliations:** Section on Translational Neuroscience, Eunice Kennedy Shriver National Institute of Child Health and Human Development, National Institutes of Health, 9000 Rockville Pike, Bethesda, MD 20892, USA; Research Institute of Children's Hospital of Philadelphia, 502-G Abramson Research Center, 3615 Civic Center Boulevard, Philadelphia, PA 19104, USA; Section on Translational Neuroscience, Eunice Kennedy Shriver National Institute of Child Health and Human Development, National Institutes of Health, 9000 Rockville Pike, Bethesda, MD 20892, USA; Center for Gene Therapy, Nationwide Children’s Hospital, Abigail Wexner Research Institute, and Department of Pediatrics, The Ohio State University College of Medicine, 575 Children's Crossroad, Columbus, OH 43215, USA; Department of Pediatrics, Vagelos College of Physicians & Surgeons, Columbia University, 630 West 168th Street, Room 10-451, New York, NY 10032, USA

**Keywords:** Choroid plexus, Lysosome, Adeno-associated virus, Alpha-mannosidosis

## Abstract

The choroid plexuses (CP) are highly vascularized structures that project into the ventricles of the vertebrate brain. The polarized epithelia of the CP produce cerebrospinal fluid by transporting water and ions into the ventricles from the blood and normally secrete a large number of proteins. We assessed the feasibility of selective CP transduction with recombinant adeno-associated virus (rAAV) gene therapy vectors for treatment of lysosomal storage disease (LSD), a broad category of neurometabolic illness associated with significant burdens to affected patients and their families. There are no ideal or complete therapeutic options currently available, especially for the central nervous system manifestations of LSDs. Alpha-mannosidosis (AMD) is an autosomal recessive prototypical LSD caused by deficiency of lysosomal alpha-mannosidase and characterized by cerebellar ataxia, neurocognitive disability, facial and skeletal abnormalities, hearing impairment, and mild immune deficiency. In a murine model of AMD, we compared the biochemical effects of CSF-directed rAAV serotypes 1, 4, 5, 6, and 9. Recombinant AAV1 and rAAV6, two closely related serotypes whose capsid sequences differ by only six amino acids, showed the most robust transduction of CP in mouse brain, consistent with their transduction of CPE in nonhuman primates and cats, as well as in other structures. We found restoration of LAMAN enzyme activity comparable to or higher than AMD heterozygote levels in the brain globally (olfactory bulb, cortex, cerebellum, brainstem). Further IND-generating preclinical experiments will advance rAAV6-LAMAN, which appears to be the most promising choroid plexus-targeting candidate serotype for future clinical translation to treat AMD.

## Introduction

Lysosomes are organelles that function as the primary digestive units within cells, and specific enzymes within lysosomes normally break down nutrients. However, patients with disorders of lysosomal storage are unable to metabolize these nutrients, resulting in extensive pathologic effects, diminished lifespans, and reduced quality of life. Cerebrospinal fluid (CSF)-directed enzyme replacement has shown promise for several LSDs [1–11] but requires repeated intrathecal administration due to short enzyme half-lives. Transplantation with native or genetically-modified hematopoietic stem cells has also been carried out for other LSDs in which there is neurological involvement, however this approach requires bone marrow ablation and can be associated with significant morbidity and mortality [12–14]. Furthermore, many LSD patients who are transplanted do not attain normal neurodevelopment, related to inefficient delivery to the CNS [15].

The choroid plexuses (CP) are highly vascularized structures that project into the ventricles of the vertebrate brain. The polarized epithelia of the CP produce cerebrospinal fluid by transporting water and ions into the ventricles from the blood and normally secrete a large number of proteins. In comparison to enzyme replacement strategies, one-time administration of recombinant adeno-associated virus (rAAV)-mediated gene transfer of missing lysosomal enzymes to choroid plexus epithelia (CPE) would be safer and more efficient. In theory, remodeling of the CPE would enable continuous synthesis and secretion of enzymes directly into the CSF with penetration to cerebral and cerebellar structures.

**Figure 1:**
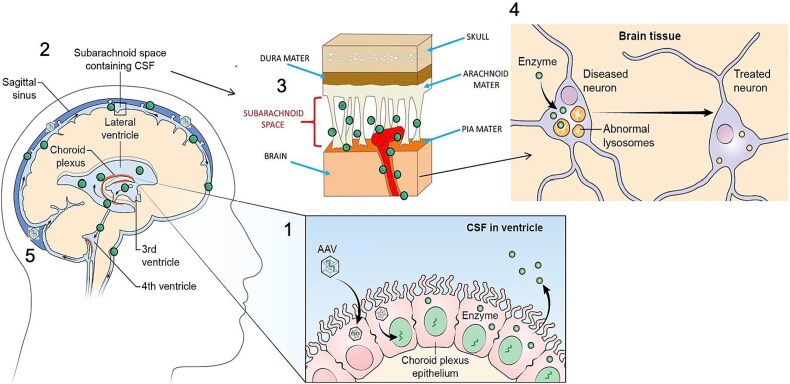
**Remodeling choroid plexus epithelia provides a powerful approach to treatment of lysosomal storage diseases.** Choroid plexus epithelia can secrete a steady supply of missing lysosomal enzymes into the CSF for distribution to diseased neurons throughout the brain, attaining global brain treatment. If successful this approach could be an ideal platform for numerous other LSDs.

CPE are post-mitotic and do not turnover [16–18], and rAAV transduction of non-dividing cells results in sustained episomal (non-integrating) transgene expression [19]. CSF flow extends into the subarachnoid fluid compartment from where proteins can ultimately reach the entire brain (Fig. 1) via Virchow-Robin spaces, paravascular CSF-filled canals surrounding perforating arteries in the brain parenchyma [20–22]. The metabolic cross-correction phenomena [23–25] in which lysosomal enzymes from enzyme-competent cells can be taken up by enzyme-deficient cells (via mannose-6-phosphate receptors) affords another key advantage to this approach. Vector leakage from the CSF to the blood, probably via bulk transport, can enable off-target rAAV transduction of peripheral organs, especially the liver, with the consequence of enhanced systemic lysosomal enzyme levels, a further advantage [26, 27].

Here, we evaluate the use of rAAV gene transfer technology to harness the potential of the choroid plexus as a long-term source of lysosomal enzyme production after a single treatment in a mouse model of a prototypical LSD, alpha-mannosidosis (AMD). The model features a distinctive biochemical phenotype, profound LAMAN deficiency, although no overt clinical manifestations or reduced lifespan are evident [28].

## Materials and methods

*Mice*: Breeding pairs of the mouse model for AMD [28] were received from Kiel University, Germany (Dr Paul Saftig), and their use was approved by the NICHD Animal Care and Use Committee.

*Intracerebroventricular injections:* Injections were performed with a 5 μl Hamilton syringe fitted with a custom needle (32 gauge, 9.5 mm long, point 4) on day two of life. For rAAV injections, 5 μl of virus (5 × 10^9^ or 5 × 10^10^ vg/5 μl) in lactated Ringer’s was injected bilaterally 2.5 mm anterior to bregma and 2.0 mm lateral to the midline, as previously described [29]. Cryoanesthesia (placing the mice on wet ice for several minutes) was used and pups were warmed post-procedure by placement on 4” × 4” infant heel warmers (Cardinal Health, Waugekan, Il).

*LAMAN activity assay:* For determination of the LAMAN activity in the brain extracts, 10ul of extract was incubated in 50ul of 0.2 M sodium citrate pH 4.4, 0.08% NaN3, 0.4% BSA, 0.15% NaCl, and 10 mM p-nitrophenyl-a-mannopyranoside as substrate for 1 h at 37°C. 200ul of 0.4 M glycine/NaOH, pH 10.4 was added to stop the reaction. Absorbance was read at 405 nm and activity expressed as nmol/mg/hr. All determinations were performed in triplicate with the appropriate negative controls (blanks).

*Immunohistochemistry*: Mice were anesthetized, decapitated, and their brains dissected and divided sagittally into right and left hemispheres, which were fixed in 10% formalin and dehydrated in 70% ethanol. Paraffin-embedded tissues were cut to a thickness of 4 μm, placed on Probe-On Plus slides (Fisher Scientific, Pittsburgh, PA), and de-paraffinized. Immunostaining for slides and controls was performed with rabbit anti-GFAP (1:100, Biocare Medical, Pacheco, CA) or mouse anti-GFP clone JL-8 for one hour (1:2000, Clontech, Mountain View, CA).

*Reverse Transcriptase-Polymerase Chain Reaction*: Total RNA in brain samples from AAV5-treated and untreated mice was extracted using RNeasy Lipid Tissue MiniKit (Qiagen). First strand cDNA synthesis was performed using Enhanced Avian TR First Strand Synthesis Kit (Sigma, St Louis, MO). PCR was used to detect LAMAN transgene and products analyzed on a 1.5% agarose gel.

*Western blotting:* Fibroblasts were collected in lysis buffer and proteins electrophoresed through SDS polyacrylamide gels (Invitrogen), transferred to polyvinylidene fluoride membranes, and incubated with mouse monoclonal antibodies and anti-mouse IgG horseradish peroxidase. Membranes were developed using SuperSignal West Pico Luminol/Enhancer Solution (Pierce).

*Statistical analysis*: Two-tailed p values were obtained for data from experiments performed in triplicate by using GraphPad software to perform student t-tests or chi square analyses.

## Results

### Transduction of choroid plexus epithelia by recombinant AAV serotypes

**Figure 2:**
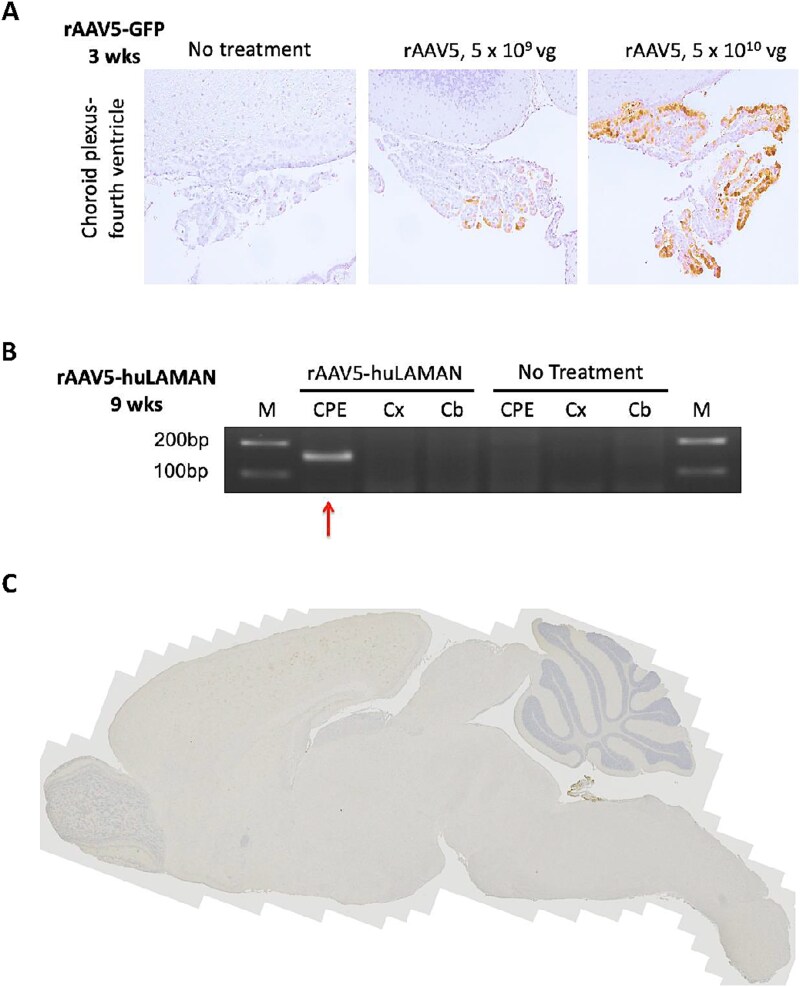
Recombinant AAV5 shows dose-dependent choroid plexus transduction in mouse brain. A. Immunohistochemical staining of AMD mouse brains three weeks after CSF-directed treatment with 5 × 10^9^ or 5 × 10^10^ rAAV5-GFP, or no treatment. rAAV5 with a GFP transgene injected into the lateral brain ventricles of AMD mice transduces CPE in dose-dependent manner. 20X magnification shown. B. RT-PCR of dissected AMD mouse brain using human LAMAN specific primers nine weeks after treatment with 5 × 10^9^ rAAV5-LAMAN, or no treatment. The LAMAN-specific product was detected only in the choroid plexus sample of rAAV treated mouse brain (arrow). CPE, choroid plexus epithelia; cx, cortex; Cb, cerebellum. C. Low power view of entire brain showing no substantial GFP immunohistochemical distribution except for choroid plexus epithelia three weeks after 5 × 10^10^ rAAV5-GFP.

Based on our prior studies of intracerebroventricular rAAV5 administration in neonatal mice that demonstrated selective transduction of choroid plexus epithelia (CPE) [29, 30], we initially explored the rAAV dose dependency of this phenomenon. Using a green fluorescent protein (GFP) reporter gene, we documented a notable increase in gene expression in animals receiving a dose of 5 × 10^10^ vg rAAV5-GFP compared to 5 × 10^9^ vg (Fig. 2A). In mice that received rAAV5-huLAMAN, we confirmed selective CPE transduction by RT-PCR of dissected brains two months after 5 × 10^9^ vg vector administration (Fig. 2B). There was no substantial GFP immunohistochemical distribution in the brain globally except for choroid plexus epithelia 3 weeks of age after rAAV5-GFP (Fig. 2C).

**Figure 3:**
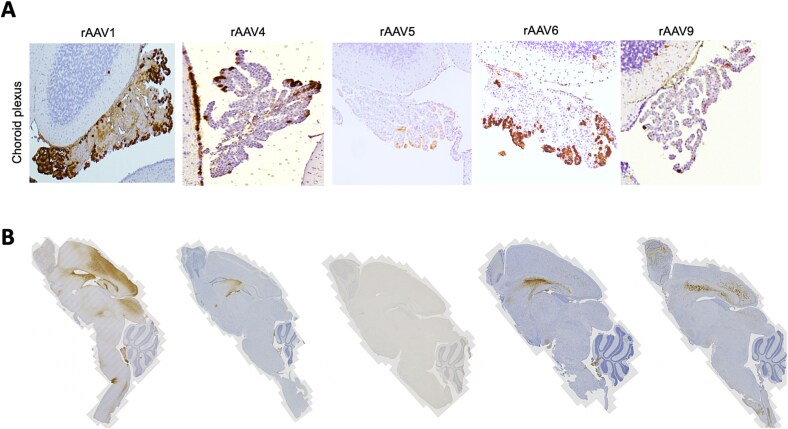
**Comparative GFP expression in mouse choroid plexus epithelia.** A. Among multiple serotypes tested at the same dose (5 × 10^9^vg AAV-green fluorescent protein (GFP) administered on P2 by intracerebroventricular injection), AAV1 and AAV6 show most robust transduction of AMD mouse choroid plexus epithelia. Sections were stained with anti-GFP antibody 3 weeks post-AAV treatment. B. Variable levels of GFP transduction across all brain regions depending on AAV serotype.

We then compared levels of CPE transduction using alternative rAAV serotypes to deliver the same dose. These experiments indicated that while rAAV4, rAAV5, and rAAV9 all transduced CPE, rAAV1 and rAAV6 appeared to provide the most robust GFP transgene expression in these cells (Fig. 3A). Variable levels of GFP transduction across other brain regions were noted, depending on AAV serotype (Fig. 3B). The latter qualitative results were consistent with LAMAN activity measured a month after CSF vector administration (Table 1), except for AAV1 and AAV9 in brain cortex.

**Table 1:** LAMAN enzyme activity (mean ± SEM in nmol/mg/hr) 1-month post-AAV administration, by AAV serotype and brain region. (also see Figs. 3, 4).

Region Serotype	**Olfactory bulb**	**Cortex**	**Cerebellum**	**Brainstem**
**AAV1 (*n* = 3)**	7.53 ± 1.37	32.96 ± 7.77	14.76 ± 2.54	12.53 ± 3.17
**AAV4 (*n* = 3)**	5.8 ± 0.5	10.33 ± 1.52	8.35 ± 1.51	8.96 ± 0.74
**AAV5 (*n* = 6)**	4.68 ± 0.98	6.5 ± 0.73	5.32 ± 1.16	8.75 ± 1.17
**AAV6 (*n* = 9)**	10.24 ± 0.47	26.5 ± 3.65	24.12 ± 3.75	16.59 ± 2.5
**AAV9 (*n* = 6)**	23.49 ± 3.5	113.27 ± 25.92	5.15 ± 0.53	10.62 ± 1.73
**HT (*n* = 8)**	11.54 ± 0.77	12.04 ± 1.26	9.23 ± 0.68	14.48 ± 0.95
**WT (*n* = 19)**	30.34 ± 2.13	31.26 ± 2.29	27.55 ± 2.36	35.03 ± 2.39
**MT (*n* = 25)**	4.38 ± 0.45	4.36 ± 0.64	5.3 ± 0.57	4.99 ± 0.47

### CSF-directed rAAV increases LAMAN enzyme activity in the AMD mouse brain

**Figure 4:**
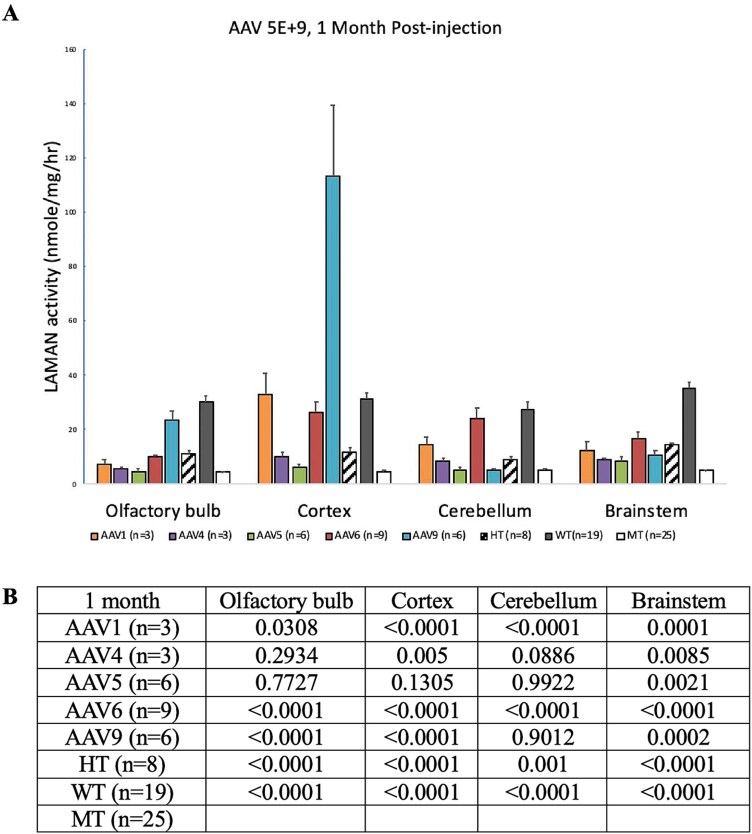
**CSF-targeted rAAVs increase brain LAMAN activity globally after a single injection.** A. LAMAN activity in wild type (WT), heterozygote (HT) or AMD mutant mouse brain regions one month after treatment with a single dose of 5 × 10^9^vg rAAV-LAMAN, or no treatment. AAV1-mediated treatment resulted in LAMAN activity equivalent or higher in AMD heterozygotes (HT) in three of four brain regions. AAV4- and AAV5-mediated delivery showed LAMAN activity not higher than HT in any brain region. AAV6-mediated delivery showed LAMAN activity equivalent or superior to HT in all four brain regions. AAV9-mediated delivery cerebellum showed markedly supranormal LAMAN activity in cortex but below HT levels in cerebellum. Error bars = SEM. B. Summary of statistical P values, by brain region and rAAV-LAMAN serotype, in comparison to untreated mutant mice.

Table 1 summarizes global brain increases in LAMAN in AMD mutant mice using the AAV serotypes we had evaluated for transduction. Untreated mutant mice (*n* = 25) showed LAMAN activity in olfactory bulb, cerebral cortex, cerebellum, and brainstem ranging from 4.36 to 5.3 nmol/mg/hr with a mean of 4.76 at one month of age (Table 1). Consistent with the CSF-directed rAAV transduction results (Fig. 3), mean LAMAN activity across mutant brain regions at the same age was higher when serotypes AAV1 or AAV6 delivered LAMAN on day 2 of life (16.9 nmol/mg/hr and 19.4 nmol/mg/hr, respectively) compared to AAV4 or AAV5 (Table 1, Fig. 4). Treatment with AAV9-LAMAN showed massive LAMAN activity increases in brain cortex (113 nmol/mg/hr) but levels in cerebellum (5.15 nmol/mg/hr) were not above untreated mutant mouse levels.

### CSF-directed rAAV sustains LAMAN enzyme activity in AMD mouse cerebellum

Based on the observed predilection for cerebellar neurological manifestations in human subjects with AMD [31], we also examined the capacity of selected rAAV serotypes to sustain cerebellar LAMAN activity over a longer time period. After administration of 5 × 10^9^ vg rAAV4, 5, 6, or 9-huLAMAN on P2, we measured LAMAN activity at two and/or six months post-treatment (Table 2). WT and untreated AMD mice were studied as controls. Two-tailed student T-test results indicated highly significant correction of LAMAN enzyme deficiency by rAAV6-LAMAN in comparison to untreated mutants at both time points (*P* = 0.0036 and 0.000006, respectively). At six months, mean cerebellar LAMAN enzyme activity was sustained at 83% wild type levels.

**Table 2:** Cerebellar LAMAN activity (mean ± SEM in nmol/mg/hr) is maintained at near normal levels after rAAV6-LAMAN injection on P2. ND: Not determined.

Treatment	2 months	6 months
rAAV4 Group size *P* value (versus untreated mutant)	8.36 ± 1.23 *n* = 3 *P* = 0.0429	ND
rAAV5 Group size P value (versus untreated mutant)	5.12 ± 0.6 *n* = 3 *P* = 0.6645	6.54 ± 2.79 *n* = 3 *P* = 0.2094
rAAV6 Group size *P* value (versus untreated mutant)	17.56 ± 6.26 *n* = 4 *P* = 0.0036	15.51 ± 1.23 *n* = 6 *P* = 0.000006
rAAV9 Group size *P* value (versus untreated mutant)	5.92 ± 0.98 *n* = 3 *P* = 0.3988	4.58 ± 1.2 *n* = 4 *P* = 0.7699
Untreated Mutant	4.68 ± 1.04 *n* = 14	5.13 ± 0.86 *n* = 15
Wild Type	22.48 ± 2.0 *n* = 13	18.69 ± 1.44 *n* = 11

### CSF-directed rAAV6-huLAMAN increases global brain LAMAN activity and transduces peripheral organs in the AMD mouse

**Figure 5:**
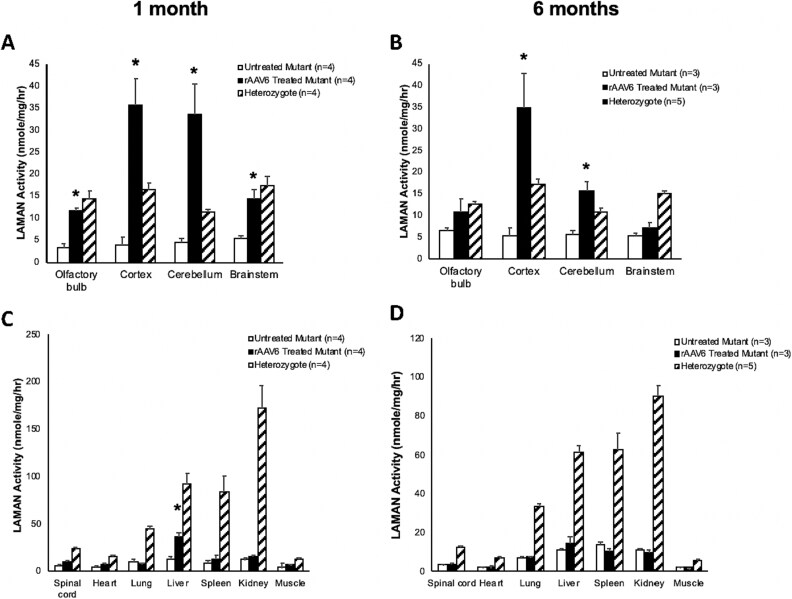
**CSF-directed rAAV6-huLAMAN in the AMD mouse model increases brain LAMAN activity and escapes to the systemic circulation and transduces peripheral organs.** LAMAN enzyme activity in heterozygote or mutant mouse brain and peripheral organs one and six months after treatment with 5 × 10^9^ vg rAAV6-huLAMAN, or no treatment. Increased LAMAN enzyme activity was detected in all four regions of the treated mouse brains compared to those of untreated mouse brain. AAV6-mediated LAMAN activity in cortex and cerebellum was sustained at least up to 6 months. LAMAN activity also increased in the liver one month after treatment, however this increased LAMAN activity was not sustained six months after treatment. Error bars represent standard error (SE) of sample means. ^*^P < 0.05.

We next conducted a series of experiments in a separate cohort to examine potential off-target effects of rAAV6-LAMAN CSF-directed administration. Following administration of 5 × 10^9^ vg rAAV6-huLAMAN, increased LAMAN enzyme activity was detected in all four regions of the treated mouse brains compared to untreated mouse brain, as expected (Fig. 5A). Statistically significant increased LAMAN activity, exceeding that in untreated heterozygotes, was sustained in cortex and cerebellum up to 6 months post-treatment (Fig. 5B). LAMAN activity in liver was statistically significantly higher one month after treatment, however by six months after treatment, hepatic LAMAN activity was not significantly higher than in untreated controls. In heterozygote mice, LAMAN activity was highest in kidney at both time points (Fig. 5C, D).

### Choroid plexus-directed rAAV6-huLAMAN improves AMD adult brain pathology

**Figure 6:**
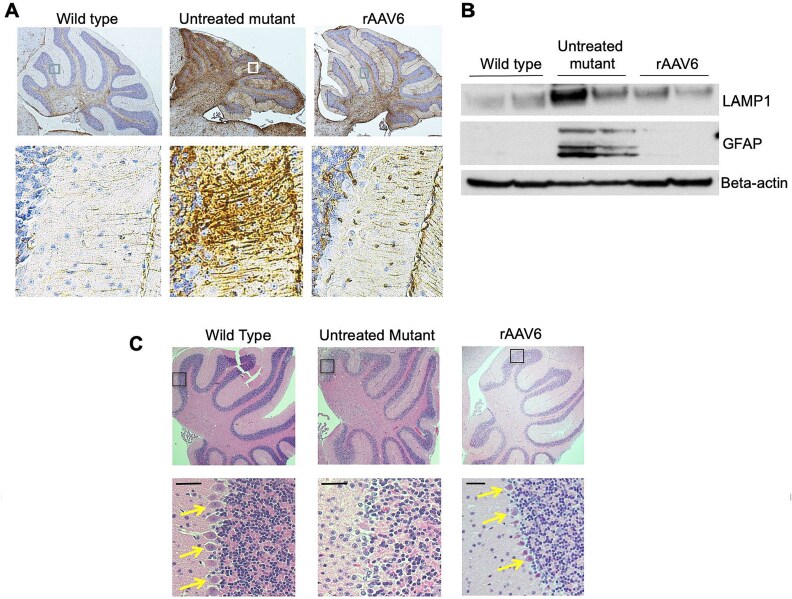
**Choroid plexus-directed rAAV6-huLAMAN prevents inflammatory response, and corrects lysosomal membrane protein abnormalities.** A. Glial fibrillary acidic protein (GFAP) staining of cerebellum in 15 month old wild type, untreated, and rAAV6-LAMAN treated AMD mouse. Untreated mutant mouse brains show markedly increased GFAP staining, a marker of astrogliosis, compared to WT and rAAV6-treated brain. Lower panels are expansions of the boxed regions in the upper panels. B. Western blot of wild type, untreated, and rAAV6-LAMAN treated AMD mouse brain homogenates from 15 month old mice. Upper and middle panels: Untreated AMD mouse brain lysates show upregulation of LAMP1 and GFAP. Normalization of LAMP1 and GFAP quantity in AMD mice is associated with rAAV6 viral gene therapy. Lower panel: Beta-actin loading control. C. H&E staining of cerebellum in 15 month old untreated/treated AMD, and wild type mice. Compared to wild type, untreated AMD brain showed significant loss of the Purkinje cell layer (arrows). Treatment with rAAV6-huLAMAN preserved the Purkinje cell layer in the mutant AMD mouse brain. Lower panels are expansions of the boxed regions in the upper panels.

We also evaluated the impact of choroid plexus-targeted AAV6-LAMAN on the inflammatory response and lysosomal membrane protein abnormalities associated with AMD in long-surviving mutant mice. Fifteen months post-treatment, notable reduction in astrogliosis were evident histochemically in the treated mouse brain as determined by glial fibrillary acidic protein (GFAP) staining of cerebellum (Fig. 6A). Similarly, western blot analyses of wild type, untreated, and rAAV6-LAMAN treated AMD mouse brain homogenates showed reduced or corrected upregulation of lysosomal associated membrane protein 1 (LAMP1) and GFAP (Fig. 6B).

We chose 15 months to evaluate brain histopathology post-treatment with rAAV6-LAMAN in order to assess evidence of long-term improvement in these known parameters of inflammation and disturbed lysosomal structure. In this mouse model, there are no overt clinical manifestations present in homozygous affected mutant mice at any age, despite the distinctive biochemical phenotype (profound LAMAN deficiency) across their lifespan. LAMP1 accumulation in one of the untreated mutants (lane 4) was less dramatic, although the inflammatory response (as measured by GFAP level) in this animal was notable (Fig. 6B, middle panel). In addition, compared to wild type, untreated AMD brains showed significant loss of the Purkinje cell layer. In contrast, treatment with rAAV6-huLAMAN preserved the Purkinje cell layer in mutant AMD mouse brain (Fig. 6C).

## Discussion

One therapeutic approach under active investigation in the field of LSD therapeutics is direct cerebrospinal fluid (CSF) administration, also called intrathecal enzyme replacement therapy (ERT). Improvements in clinical signs and/or neuropathology have been reported in several different LSD animal models using this approach, including MPS I dogs [2, 32], MPS IIIA mice and dogs [4–6], Krabbe disease mice [7], late infantile neuronal ceroid lipofuscinosis mice and dogs [8, 9], Niemann-Pick A mice [10], MPS VI cats [11]. CSF-directed ERT has also been used in human subjects with MPS I [33, 34], MPS II [35], MPS IIIA [36] and Pompe disease [37] with preliminary evidence of efficacy. These combined animal and human data offer a powerful proof of principle for our approach. CSF-directed ERT is predicted to be more effective at treating the global CNS effects of LSDs than systemic (intravenous) ERT, since traversing the blood–brain barrier is not required. In the initial systemic ERT studies in patients with alpha-mannosidosis, reduction of CSF oligosaccharides was minimal (26%) compared to the reduction in urine (54%) and blood (89%), reflecting inefficient brain delivery [38]. The European Medicines Agency’s (EMA) and US Food and Drug Administration (FDA) approved the drug (Lamzede or Velmanase alfa) for AMD in 2018 and 2023, respectively. They stated that this product does not cross the blood–brain barrier [39], as for all recombinant lysosomal enzymes approved for ERT. Studies published subsequently claimed modest improvements in AMD patients’ 3-min stair-climb tests [40, 41] and in a *post-hoc* global treatment response index [42].

Problems common to both systemic (IV) and CSF-directed ERT include short half-lives of lysosomal enzymes, the inconvenience of weekly administration, immune responses to the recombinant enzyme, and cost. For CSF-directed intrathecal ERT, additional undesirable risks include meningitis, dural inflammation, discitis, and lumbar epidural abscess as well as increased patient anxiety and discomfort related to the procedure. CSF-directed ERT has not been proposed by the developers of Velmanase alfa, presumably due to these factors.

Our rationale for choroid plexus-targeted gene therapy in this study was to complement enzyme replacement for alpha-mannosidosis with a single dose of rAAV vector into the CSF that will transduce and remodel choroid plexus epithelia to produce a steady supply of LAMAN for delivery throughout the brain, as occurs in other murine LSDs [43]. Concurrently, some peripheral therapeutic effects are also expected, via vector transport to the systemic blood circulation and off-target transduction of peripheral organs [26] (Fig. 5). The principle of CSF-directed delivery of rAAV vectors to larger animal brains is also well established [44].

The AMD mouse model we used features no overt clinical manifestations in homozygous affected mutant mice at any age, despite the distinctive biochemical phenotype of profound LAMAN deficiency across their lifespan. When we tested CSF-directed AAV5 in this AMD mouse, selective choroid plexus epithelia targeting was noted, in a dose-dependent pattern (Fig. 2). When the human LAMAN transgene was substituted for GFP, significant levels of LAMAN were produced throughout the brain globally (Tables 1, 2). These data indicate that choroid plexus epithelia transduction alone can produce and circulate normal LAMAN adequate to treat the entire mouse brain and that expression is sustained for a minimum of 6 months (human age equivalent = 20 years) [45]. Recombinant AAV6 appeared to be the most efficient serotype in this process. The AAV6 capsid is closely related to AAV1, differing only by several amino acids and both utilize the sialic acid receptor [46]. However, AAV1-associated restoration of LAMAN activity 1 month post-treatment was lower in three of four brain regions (especially in cerebellum) compared to AAV6 (Table 1, Fig. 4).

We show that LAMAN enzyme activity mediated by a single AAV6 injection to the CSF (lateral ventricles) is sustained in comparison to alternative serotypes at the same dose (5 ×10^9^vg, Table 2). Our results focusing on the diseased AMD cerebellum indicate that neonatal AAV6 treatment also markedly ameliorates reactive astroglial response (GFAP) and reduces lysosomal storage (LAMP1) compared to untreated AMD mice. LAMP1 reduction in one untreated mutants was less dramatic, although reduction of the inflammatory response (GFAP) in this animal was striking (Fig. 6).

These overall findings suggest AAV6-LAMAN as a possible candidate for an investigational new drug (IND) application in a future clinical trial of CSF-directed gene therapy for human subjects with AMD. The choroid plexus-targeted viral gene therapy approach we describe has potential for high impact on clinical practice since the largest current barriers to health for patients with AMD and other LSDs would be circumvented. Newborn screening for AMD, to afford the earliest possible detection of affected infants and children, will enhance effectiveness of both viral gene therapy and enzyme replacement treatment approaches to this illness.

## References

[1] Hemsley KM, Hopwood JJ. Delivery of recombinant proteins via the cerebrospinal fluid as a therapy option for neurodegenerative lysosomal storage diseases. Int J Clin Pharmacol Ther 2009;47:S118–S123. 20040322 10.5414/cpp47118

[2] Kakkis E, McEntee M, Vogler C. et al. Intrathecal enzyme replacement therapy reduces lysosomal storage in the brain and meninges of the canine model of MPS I. Mol Genet Metab 2004;83:163–174. 15464431 10.1016/j.ymgme.2004.07.003

[3] Dierenfeld AD, McEntee MF, Vogler CA. et al. Replacing the enzyme alpha-Liduronidase at birth ameliorates symptoms in the brain and periphery of dogs with mucopolysaccharidosis type I. Sci Transl Med 2010;2:60ra89. 10.1126/scitranslmed.3001380 PMC3075726 21123810

[4] Hemsley KM, King B, Hopwood JJ. Injection of recombinant human sulfamidase into the CSF via the cerebellomedullary cistern in MPS IIIA mice. Mol Genet Metab 2007;90:313–328. 17166757 10.1016/j.ymgme.2006.10.005

[5] Beard H, Luck AJ, Hassiotis S. et al. Determination of the role of injection site on the efficacy of intra-CSF enzyme replacement therapy in MPS IIIA mice. Mol Genet Metab 2015;115:33–40. 25795516 10.1016/j.ymgme.2015.03.002

[6] Crawley AC, Marshall N, Beard H. et al. Enzyme replacement reduces neuropathology in MPS IIIA dogs. Neurobiol Dis 2011;43:422–434. 21550404 10.1016/j.nbd.2011.04.014

[7] Lee WC, Tsoi YK, Troendle FJ. et al. Single dose intracerebroventricular administration of galactocerebrosidase improves survival in a mouse model of globoid cell leukodystrophy. FASEB J 2007;21:2520–2527. 17403939 10.1096/fj.06-6169com

[8] Chang M, Cooper JD, Sleat DE. et al. Intraventricular enzyme replacement improves disease phenotypes in a mouse model of late infantile neuronal ceroid lipofuscinosis. Mol Ther 2008;16:649–656. 18362923 10.1038/mt.2008.9

[9] Katz ML, Coates JR, Sibigtroth CM. et al. Enzyme replacement therapy attenuates disease progression in a canine model of late-infantile neuronal ceroid lipofuscinosis (CLN2 disease). J Neurosci Res 2014;92:1591–1598. 24938720 10.1002/jnr.23423 PMC4263309

[10] Dodge JC, Clarke J, Treleaven CM. et al. Intracerebroventricular infusion of acid sphingomyelinase corrects CNS manifestations in a mouse model of Niemann-pick a disease. Exp Neurol 2009;215:349–357. 19059399 10.1016/j.expneurol.2008.10.021

[11] Auclair D, Finnie J, White J. et al. Repeated intrathecal injections of recombinant human 4-sulphatase remove dural storage in mature mucopolysaccharidosis VI cats primed with a short-course tolerisation regimen. Mol Genet Metab 2010;99:132–141. 19896877 10.1016/j.ymgme.2009.10.002

[12] Sessa M, Lorioli L, Fumagalli F. et al. Lentiviral haemopoietic stem-cell gene therapy in early-onset metachromatic leukodystrophy: an ad-hoc analysis of a non-randomised, open-label, phase 1/2 trial. Lancet 2016;388:476–487. 27289174 10.1016/S0140-6736(16)30374-9

[13] Eichler F, Duncan C, Musolino PL. et al. Hematopoietic stem-cell gene therapy for cerebral Adrenoleukodystrophy. N Engl J Med 2017;377:1630–1638. 28976817 10.1056/NEJMoa1700554 PMC5708849

[14] Lawitschka A, Peters C. Long-term effects of Myeloablative allogeneic hematopoietic stem cell transplantation in Pediatric patients with acute lymphoblastic Leukemia. Curr Oncol Rep 2018;20:74. 30074106 10.1007/s11912-018-0719-5

[15] Mynarek M, Tolar J, Albert MH. et al. Allogeneic hematopoietic SCT for alpha-mannosidosis: an analysis of 17 patients. Bone Marrow Transplant 2012;47:352–359. 21552297 10.1038/bmt.2011.99

[16] McDonald TF, Green K. Cell turnover in ciliary epithelium compared to other slow renewing epithelia in the adult mouse. Curr Eye Res 1988;7:247–252. 3359810 10.3109/02713688809047029

[17] Liddelow SA, Dziegielewska KM, Vandeberg JL. et al. Development of the lateral ventricular choroid plexus in a marsupial, Monodelphis domestica. Cerebrospinal Fluid Res 2010;7:16. 20920364 10.1186/1743-8454-7-16 PMC2964622

[18] Lun MP, Monuki ES, Lehtinen MK. Development and functions of the choroid plexus-cerebrospinal fluid system. Nat Rev Neurosci 2015;16:445–457. 26174708 10.1038/nrn3921 PMC4629451

[19] Deverman BE, Ravina BM, Bankiewicz KS. et al. Gene therapy for neurological disorders: progress and prospects. Nat Rev Drug Discov 2018;17:641–659. 30093643 10.1038/nrd.2018.110

[20] Szentistványi I, Patlak CS, Ellis RA. et al. Drainage of interstitial fluid from different regions of rat brain. Am J Phys 1984;246:F835–F844. 10.1152/ajprenal.1984.246.6.F835 6742132

[21] Rennels ML, Gregory TF, Blaumanis OR. et al. Evidence for a ‘paravascular’ fluid circulation in the mammalian central nervous system, provided by the rapid distribution of tracer protein throughout the brain from the subarachnoid space. Brain Res 1985;326:47–63. 3971148 10.1016/0006-8993(85)91383-6

[22] Iliff JJ, Wang M, Liao Y. et al. A paravascular pathway facilitates CSF flow through the brain parenchyma and the clearance of interstitial solutes, including amyloid β. Sci Transl Med 2012;4:147ra111. 10.1126/scitranslmed.3003748 PMC3551275 22896675

[23] Fratantoni JC, Hall CW, Neufeld EF. Hurler and Hunter syndromes: mutual correction of the defect in cultured fibroblasts. Science 1968;162:570–572. 4236721 10.1126/science.162.3853.570

[24] Watson DJ, Wolfe JH. Lentiviral vectors for gene transfer to the central nervous system. Applications in lysosomal storage disease animal models. Methods Mol Med 2003;76:383–403. 12526176 10.1385/1-59259-304-6:383

[25] Biffi A . Gene therapy for lysosomal storage disorders: a good start. Hum Mol Genet 2016 2016;25:R65–R75. 10.1093/hmg/ddv457 26604151

[26] Haddad MR, Choi EY, Zerfas PM. et al. Cerebrospinal fluid- directed rAAV9-rsATP7A plus subcutaneous copper Histidinate advance survival and outcomes in a Menkes disease mouse model. Mol Ther Methods Clin Dev 2018;10:165–178. 30090842 10.1016/j.omtm.2018.07.002 PMC6080355

[27] Karolewski BA, Wolfe JH. Genetic correction of the fetal brain increases the lifespan of mice with the severe multisystemic disease mucopolysaccharidosis type VII. Mol Ther 2006;14:14–24. 16624622 10.1016/j.ymthe.2006.02.012

[28] Stinchi S, Lüllmann-Rauch R, Hartmann D. et al. Targeted disruption of the lysosomal alpha-mannosidase gene results in mice resembling a mild form of human alpha-mannosidosis. Hum Mol Genet 1999;8:1365–1372. 10400983 10.1093/hmg/8.8.1365

[29] Donsante A, Yi L, Zerfas P. et al. ATP7A gene addition to the choroid plexus results in long-term rescue of the lethal copper transport defect in a Menkes disease mouse model. Mol Ther 2011;19:2114–2123. 21878905 10.1038/mt.2011.143 PMC3242653

[30] Watson DJ, Passini MA, Wolfe JH. Transduction of the choroid plexus and ependyma in neonatal mouse brain by vesicular stomatitis virus glycoprotein-pseudotyped lentivirus and adeno-associated virus type 5 vectors. Hum Gene Ther 2005;16:49–56. 15703488 10.1089/hum.2005.16.49

[31] Malm D, Nilssen Ø. Alpha-Mannosidosis. 2001 Oct 11 [updated 2019 Jul 18]. In: Adam M.P., Mirzaa G.M., Pagon R.A.. et al. (eds.), GeneReviews®[Internet]. Seattle (WA): University of Washington, Seattle, 1993–2023.

[32] Dierenfeld AD, McEntee MF, Vogler CA. et al. Replacing the enzyme alpha-L-iduronidase at birth ameliorates symptoms in the brain and periphery of dogs with mucopolysaccharidosis type I. Sci Transl Med 2010;2:60ra89 PMCID: PMC3075726. 10.1126/scitranslmed.3001380 PMC3075726 21123810

[33] Munoz-Rojas M-V, Vieira T, Costa R. et al. Intrathecal enzyme replacement therapy in a patient with mucopolysaccharidosis type I and symptomatic spinal cord compression. Am J Med Genet A 2008;146A:2538–2544. 18792977 10.1002/ajmg.a.32294

[34] Dickson PI, Kaitila I, Harmatz P. et al. Mucopolysaccharidosis I intrathecal research collaborative. Safety of laronidase delivered into the spinal canal for treatment of cervical stenosis in mucopolysaccharidosis I. Mol Genet Metab 2015;116:69–74. 26260077 10.1016/j.ymgme.2015.07.005 PMC4572891

[35] Muenzer J, Hendriksz CJ, Fan Z. et al. A phase I/II study of intrathecal idursulfase-IT in children with severe mucopolysaccharidosis II. Genet Med 2016;18:73–81. 25834948 10.1038/gim.2015.36

[36] Jones SA, Breen C, Heap F. et al. A phase 1/2 study of intrathecal heparan-N-sulfatase in patients with mucopolysaccharidosis IIIA. Mol Genet Metab 2016;118:198–205. 27211612 10.1016/j.ymgme.2016.05.006

[37] Hordeaux J, Dubreil L, Robveille C. et al. Long-term neurologic and cardiac correction by intrathecal gene therapy in Pompe disease. Acta Neuropathol Commun 2017;5:66. 28874182 10.1186/s40478-017-0464-2 PMC5585940

[38] Borgwardt L, Dali CI, Fogh J. et al. Enzyme replacement therapy for alpha-mannosidosis: 12 months follow-up of a single Centre, randomised, multiple dose study. J Inherit Metab Dis 2013;36:1015–1024. 23494656 10.1007/s10545-013-9595-1

[39] Ceccarini MR, Codini M, Conte C. et al. Alpha-Mannosidosis: therapeutic strategies. Int J Mol Sci 2018;19. 10.3390/ijms19051500 PMC5983820 29772816

[40] Lund AM, Borgwardt L, Cattaneo F. et al. Comprehensive long-term efficacy and safety of recombinant human alpha-mannosidase (velmanase alfa) treatment in patients with alpha-mannosidosis. J Inherit Metab Dis 2018;41:1225–1233. 29725868 10.1007/s10545-018-0175-2 PMC6326957

[41] Borgwardt L, Guffon N, Amraoui Y. et al. Efficacy and safety of Velmanase alfa in the treatment of patients with alpha- mannosidosis: results from the core and extension phase analysis of a phase III multicentre, double-blind, randomised, placebo-controlled trial. J Inherit Metab Dis 2018;41:1215–1223. 29846843 10.1007/s10545-018-0185-0 PMC6326984

[42] Harmatz P, Cattaneo F, Ardigò D. et al. Enzyme replacement therapy with velmanase alfa (human recombinant alpha-mannosidase): novel global treatment response model and outcomes in patients with alpha-mannosidosis. Mol Genet Metab 2018;124:152–160. 29716835 10.1016/j.ymgme.2018.04.003

[43] Passini MA, Watson DJ, Vite CH. et al. Intraventricular brain injection of adeno-associated virus type 1 (AAV1) in neonatal mice results in complementary patterns of neuronal transduction to AAV2 and total long-term correction of storage lesions in the brains of β-Glucuronidase-deficient mice. J Virol 2003;77:7034–7040. 12768022 10.1128/JVI.77.12.7034-7040.2003 PMC156185

[44] Hunter JE, Molony CM, Bagel JH. et al. Transduction characteristics of alternative adeno-associated virus serotypes in the cat brain by intracisternal delivery. Mol Ther Methods Clin Dev 2022;26:384–393. 36034772 10.1016/j.omtm.2022.07.007 PMC9391516

[45] Dutta S, Sengupta P. Men and mice: relating their ages. Life Sci 2016;152:244–248. 26596563 10.1016/j.lfs.2015.10.025

[46] Huang LY, Patel A, Ng R. et al. Characterization of the adeno-associated virus 1 and 6 sialic acid binding site. J Virol 2016;90:5219–5230. 26962225 10.1128/JVI.00161-16 PMC4934739

